# Editorial: Accelerated Brain Aging: Different Diseases—Different Imaging Patterns

**DOI:** 10.3389/fneur.2022.889538

**Published:** 2022-04-07

**Authors:** Dusko B. Kozic, Majda M. Thurnher, Jasmina Boban, Pia C. Sundgren

**Affiliations:** ^1^Faculty of Medicine, University of Novi Sad, Novi Sad, Serbia; ^2^Oncology Institute of Vojvodina, Sremska Kamenica, Serbia; ^3^Department for Biomedical Imaging and Image-Guided Therapy, Medical University of Vienna, Vienna, Austria; ^4^Department of Radiology, Faculty of Medicine, University of Novi Sad, Novi Sad, Serbia; ^5^Department of Medical Imaging and Physiology, Skane University Hospital, Lund, Sweden; ^6^Department of Diagnostic Radiology, Clinical Sciences, Lund University, Lund, Sweden

**Keywords:** brain aging, brain, magnetic resonance imaging (MRI), functional magnetic brain imaging (fMRI), brain disease

With a significantly prolonged lifespan, modern societies are facing difficulties associated with impaired cognitive function and subsequent difficulties in the everyday life of the elderly population. Neurobiological changes in healthy aging affect the functional organization of the brain and cognitive abilities including working memory, processing speed, and executive functions (1). Additionally, subtle age-related dysfunction in the regional brain integrity, as well as age-related differences in functional and structural connectivity, can be observed (2).

Besides pathological processes that directly affect neurons and result in accelerated brain aging (multiple sclerosis, traumatic brain injury, cerebral amyloid angiopathy, psychiatric disorders), many systemic disorders are also related to a certain degree of cognitive disabilities (hypothyroidism, diabetes, connective tissue disorders, infections hyperlipidemia, hypertension, obesity and autoimmune diseases, and many others) (3–7). Timely recognition and awareness of the presence of chronic neuronal injury in these disorders are important in order to identify the individuals at risk and to take preventive measures to maintain the quality of life.

The aim of this Research Topic was to discuss the most advanced knowledge in the field of imaging of healthy and accelerated brain aging. In addition, with special attention directed to the emerging advanced imaging techniques, applied to enable a better understanding of the evolution of brain atrophy, and structural and functional impairment during physiological brain aging.

Considering healthy aging, a special focus was drawn to gender difference, which are presented in some papers. In the paper by Podgorski, Bladowska, et al. differences in brain aging between females and males were evaluated using novel volumetric and surface-based indices obtained by magnetic resonance imaging (MRI), concluding that brain aging in both genders starts at a similar age. However, it seems that there is a greater cortical loss in age-matched females, with relative sparing of white matter, following a complex pattern of cortical remodeling. A companion paper by Podgorski, Waliszewska-Prosół, et al. showed that there is increased resting-state functional connectivity in default mode network observed on resting-state fMRI could serve as a compensatory mechanism for the above-mentioned cortical loss and remodeling in elderly females. Kavroulakis et al. demonstrated age-related intrinsic connectivity reductions and hemodynamic changes in default mode network in parietal regions, associated with impaired neurovascular coupling. Their paper supported the retrogenesis hypothesis with these age-related reductions in connectivity being greater in anterior compared to posterior cortices. Furthermore, the authors showed that these changes were reflected in self-reported depression symptoms, also increasing with advancing age. The importance of epidemiological and socio-economic factors in healthy brain aging was tackled in the paper by Jacków-Nowicka et al. who demonstrated that reduction in the gray matter volume was strongly associated with the accumulation of non-specific white matter hyperintensities that seem to be potentially preventable by maintaining normal blood pressure and cholesterol levels.

Summarizing the articles that elaborated on healthy brain aging and recent results speak in favor of gender specificity as well as the certain potential of functional remodeling of brain connectivity expressed as a salvage mechanism for overcoming cortical volume loss, demonstrated mostly in default mode network. The impact of an individual's lifestyle must not be neglected, given that the burden of white matter hyperintensities (partly associated with arterial hypertension and hypercholesterolemia) adds to the reduction of gray matter volume.

Benbrika et al. summarized the clinical and imaging data in the follow-up of extra-motor manifestations in amyotrophic lateral sclerosis. They demonstrated that little deterioration occurred in extra-motor manifestations and psychological state even though progressive cortical thinning was detected on imaging. However, progressive deterioration in mental flexibility, executive functions, and inhibition abilities was observed, associated with a progressive cortical reduction in prefrontal areas.

Mansoor et al. showed the promising potential of automated segmentation in evaluating caudate atrophy in Huntington disease, suggesting that fully automated pipelines could be used to generate outcome measures for clinical trials. Dal-Bianco et al. observed the role of iron-rim in patients with multiple sclerosis on 7T. They observed that patients with present iron rim showed also higher FLAIR lesion counts, smaller thalamic volumes, and higher serum neurofilament concentrations.

However, preliminary data showed no significant difference in the neurocognitive performance of patients with and without iron rim in a 1-year follow-up period.

Finally, a pilot study by Zhang et al. showed a rather promising role of a new PET tracer in cerebral amyloid angiopathy, since it seems, according to the pilot study, that the [68Ga] Ga-P14-032 PET probe binds preferentially to vascular amyloid.

Acceleration of brain aging in some neurological and systemic disorders remains to be a dynamic field of investigation, especially with the development of modern imaging techniques and hybrid imaging. However, a clear understanding of healthy brain aging is crucial for diagnosis, accurate characterization, and monitoring of neurodegenerative processes. Modern techniques of neuroimaging are able to provide some imaging biometrics and markers of healthy and accelerated brain aging, and to improve our comprehension of physiological changes in brain parenchyma and their reflection on imaging. In line with the better understanding and growth in the field of imaging, machine learning and brain aging models are gaining more and more attention. Age prediction modeling is in progress but there are many features of these models that yet must be explored, especially in the means of specificity. In other words, the detection of the gap between chronological and predicted age currently does not offer the clear direction of further investigation of specific underlying pathological process. Additionally, emerging evidence of (not only) sex differences in the process of brain aging, presented also in this Research Topic, makes choosing the right classifier even more complicated. To conclude, large population studies combined with the inclusion of epidemiologic and socioeconomic data in the mathematical and machine learning models are the directions in which future studies on accelerated brain aging should be driven.

## Author Contributions

All authors listed have made a substantial, direct, and intellectual contribution to the work and approved it for publication.

## Conflict of Interest

The authors declare that the research was conducted in the absence of any commercial or financial relationships that could be construed as a potential conflict of interest.

## Publisher's Note

All claims expressed in this article are solely those of the authors and do not necessarily represent those of their affiliated organizations, or those of the publisher, the editors and the reviewers. Any product that may be evaluated in this article, or claim that may be made by its manufacturer, is not guaranteed or endorsed by the publisher.
